# Mean Bias in Seasonal Forecast Model and ENSO Prediction Error

**DOI:** 10.1038/s41598-017-05221-3

**Published:** 2017-07-20

**Authors:** Seon Tae Kim, Hye-In Jeong, Fei-Fei Jin

**Affiliations:** 1 Climate Prediction Department, APEC Climate Center, Busan, South Korea; 2 Department of Atmospheric Sciences, University of Hawaii at Manoa, Honolulu, HI USA

## Abstract

This study uses retrospective forecasts made using an APEC Climate Center seasonal forecast model to investigate the cause of errors in predicting the amplitude of El Niño Southern Oscillation (ENSO)-driven sea surface temperature variability. When utilizing Bjerknes coupled stability (BJ) index analysis, enhanced errors in ENSO amplitude with forecast lead times are found to be well represented by those in the growth rate estimated by the BJ index. ENSO amplitude forecast errors are most strongly associated with the errors in both the thermocline slope response and surface wind response to forcing over the tropical Pacific, leading to errors in thermocline feedback. This study concludes that upper ocean temperature bias in the equatorial Pacific, which becomes more intense with increasing lead times, is a possible cause of forecast errors in the thermocline feedback and thus in ENSO amplitude.

## Introduction

Long-term ocean memory allows seasonal forecast models to provide a few season-ahead skillful predictions of sea surface temperature (SST) variability associated with the El Niño-Southern Oscillation (ENSO) in the tropical Pacific Ocean^1^. ENSO is the most dominant natural climate fluctuation on interannual time scales and has an enormous impact on global climate^2^. Moreover, skillful prediction of the ENSO-driven SST variability makes it possible to provide reliable global seasonal climate prediction of variables, e.g., precipitation and surface temperature^3–5^. Therefore, successful ENSO prediction with long lead times gives decision makers an opportunity to consider predicted climate anomalies to reduce the socioeconomic and environmental impacts of ENSO.

There has been noticeable progress in the prediction of ENSO events using coupled climate models^6, 7^. The recent decade’s coupled models perform better than statistical models^7^. However, coupled models still have systematic errors in the tropical Pacific climatological mean states. Previous inter-model comparison studies attempted to reveal the relationship between the tropical Pacific mean state and ENSO simulations and highlighted the importance of properly simulating the climatological mean state in the tropical Pacific for realistic simulations of ENSO^8–10^. Specifically, the climatological cold bias in the upper tropical Pacific Ocean affects the reliability of simulating the amplitude of ENSO-driven SST variability, leading to an inter-model diversity of ENSO representations^11^.

During coupled model integrations, model drift, where the climate state of the coupled model tends to move toward the model’s own climatology (which is biased from observations), is inevitable^12, 13^. The enhanced model errors regarding the tropical Pacific mean states during forecast runs could affect the predictive capability for ENSO events. Some studies have shown that an improved representation of ENSO variability can be obtained when using a correction method to reduce the tropical Pacific mean states^14^. To improve ENSO prediction with coupled models, some studies have attempted to understand the source and reason of the mean state biases with hindcast simulations because one can trace the development of the climatological mean bias in the tropical Pacific during model integrations^13, 15^. However, no study has yet investigated where the mean state errors start and grow or how growing mean state errors dynamically affects the ENSO prediction capability of coupled models as forecast lead times increase. By utilizing hindcast simulations, we can explore how the growing biases are dynamically connected to the errors in ENSO prediction.

The APEC Climate Center (APCC) has made great efforts to provide reliable forecast information for the Asia-Pacific region by operating the APCC multi-model ensemble (MME) forecast system (www.apcc21.org for more information). By developing a simple initialization scheme based on coupled nudging, the APCC operationalized the National Center for Atmospheric Research (NCAR) Community Climate System Model version 3 (CCSM3)^16^ to support the APCC MME system. The APCC seasonal forecast model is called the APCC CCSM3.

Using hindcast simulations of the APCC CCSM3, this study aims to understand how upper ocean mean temperature bias in the tropical Pacific, which intensifies with increasing forecast lead time, is associated with errors in ENSO-related feedbacks and hence errors in ENSO amplitude forecasts.

## Results

### ENSO forecast errors in the APCC CCSM3

**Figure 1:**
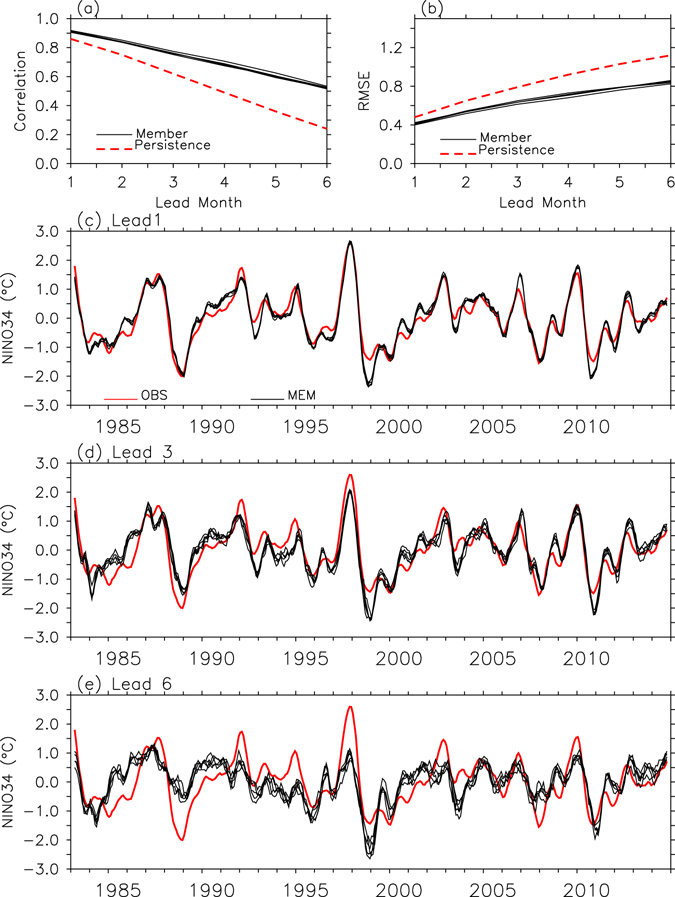
Prediction skill of ENSO. (**a**) Anomaly temporal correlation coefficient (TCC), (**b**) root mean square error (RMSE) between observed and predicted Niño 3.4 indices from 1- to 6-month lead times, and time series of Niño 3.4 index from observations (red line) and forecasts (black lines) at (**c**) 1-month, (**d**) 3-month, and (**e**) 6-month lead times. In (**a**,**b**), TCCs and RMSEs of persistence and individual member predictions are indicated by red dashed lines and black solid lines, respectively. In (**c**–**e**), for a clear comparison, the 3-month running mean is applied to the Niño 3.4 index before plotting.

To examine the overall ability of the APCC CCSM3 to predict ENSO-driven SST variability, the anomaly temporal correlation coefficient (TCC) and the root mean square error (RMSE) between the observed Niño 3.4 index and the index from individual forecasts were calculated, as shown in Fig. 1a and b. It is evident that the model forecasts of the Niño 3.4 index have larger TCCs than the persistence forecasts over forecast lead times. Here, persistence forecasts are made by assuming that the observed SST anomaly for the month prior to the initial time persists without change over the forecast period. The average of the TCCs for the Niño 3.4 index prediction from individual members at an early lead time (i.e., 1-month lead) is 0.91, and for 5-month lead forecasts the averaged TCC is 0.61, indicating that the APCC CCSM3 model can make skillful predictions of interannual ENSO events to the 5-month lead times even with the simple wind-ocean temperature nudging initialization method. Even at a 6-month lead time, the ENSO prediction has statistically significant TCC scores at the 99% level (according to Student’s t-test), the average of which is 0.51. Furthermore, the RMSEs of the predictions of the Niño 3.4 index are smaller than those of the persistence forecasts.

**Figure 2:**
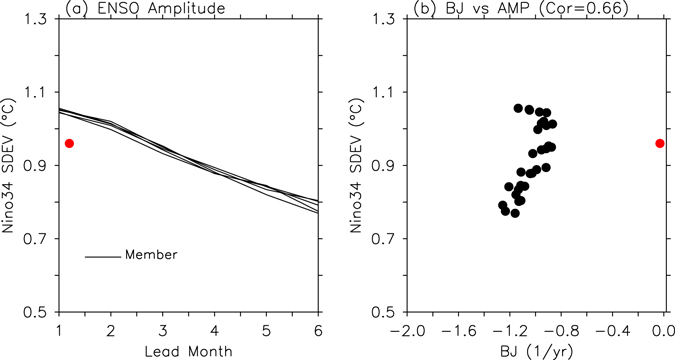
Error in ENSO amplitude forecasts. (**a**) ENSO amplitude (°C) and (**b**) scatter plots of ENSO amplitude versus BJ index from 1- to 6-month lead times. Black lines and black dots correspond to individual forecasts. Red dots indicate observations. In (**b**), a correlation coefficient between BJ index and ENSO amplitude from individual forecasts is shown.

If we examine the time series of the Niño 3.4 index from the model predictions (Fig. 1c) more closely, the predicted Niño 3.4 indices are close to observations at a 1-month lead time. However, when the lead time increases, the inter-ensemble member spread also tends to increase. In addition to the spread, it is evident that the amplitude of the Niño 3.4 index, for both the El Niño and La Niña phases, is reduced with an increase in the forecast lead time. The increased ENSO amplitude forecast errors with increasing forecast lead times can be clearly identified in Fig. 2a, where it can be seen that the ENSO amplitude gradually decreases and eventually reaches a magnitude (0.59~0.65 °C) that is much smaller than that of the observed level (0.96 °C) at a 6-month lead time. Here, the amplitude of the ENSO SST variability was defined as the standard deviation of the Niño 3.4 index.

### ENSO forecast errors and mean state bias

In this study, the Bjerknes coupled stability (BJ) index (see methods) is used to understand the possible causes of ENSO amplitude forecast errors^10, 17^. The BJ index formula was developed to assess the overall stability of the coupled ENSO mode. Its capability to estimate ENSO stability was successfully verified through a detailed eigen-analysis of an intermediate-coupled model^10^ and through various simulations of a hybrid-coupled model with different atmospheric and oceanic mean state conditions^17^. The BJ index has been utilized in many studies for revealing the possible causes of changes in ENSO behavior under different mean states^11, 18–23^. The BJ index can estimate the linear ENSO growth rate derived from a coupled system of observations and climate models, and hence it is essentially related to ENSO amplitude. Furthermore, as ENSO-related feedbacks can be partitioned by the BJ index analysis, it is possible to understand errors in the atmosphere-ocean coupling process associated with ENSO amplitude forecast errors.

As mentioned in the Methods section, we reconstructed monthly datasets from forecasts at 1- to 6-month lead times, and each forecast has five ensemble members. Therefore, we obtained 30 samples each for ENSO amplitude and the BJ index and its related terms (i.e., feedback terms and the feedback-related response sensitivity coefficients and mean state terms). Using these samples, correlation and regression analyses were performed.

As found in scatterplots of ENSO amplitude versus the BJ index (Fig. 2b) from the individual forecasts, the growth rates estimated using the BJ index have a significant correlation coefficient of 0.66 with ENSO amplitude. This indicates that the BJ index of hindcast simulations tends to become more negative with an increase in forecast lead time, and hence suggests that the BJ index analysis can provide an insight into the reason behind ENSO amplitude forecasting errors based on ENSO dynamics.

In Fig. 2b, one can see that the BJ index from the 1-month lead time forecast (−1.02 ± 0.08 yr^−1^) is more negative than the BJ index from observations (−0.03 yr^−1^), whereas ENSO amplitude (1.1 °C for all members) at the 1-month lead time is slightly larger than that from the observations. This discrepancy is because the BJ index formula does not consider the possible role of non-linearity, noise, and seasonal cycles that also contribute to ENSO amplitude^24–26^. A recent study comparing BJ index terms with direct heat budget terms indicated that non-linearity is not captured by the BJ index^27^. However, numerous studies have shown that ENSO dynamics are largely linear^8, 10, 28^, as supported by the success of the ENSO oscillator theories^8, 28^. The BJ index formula is intended to capture this intrinsic nature of ENSO linear dynamics^10^. Despite its shortcomings, the significant correlation between the ENSO amplitude and the BJ index strongly indicates that we can still utilize BJ index analysis to reveal the reason for ENSO amplitude forecast errors.

**Figure 3:**
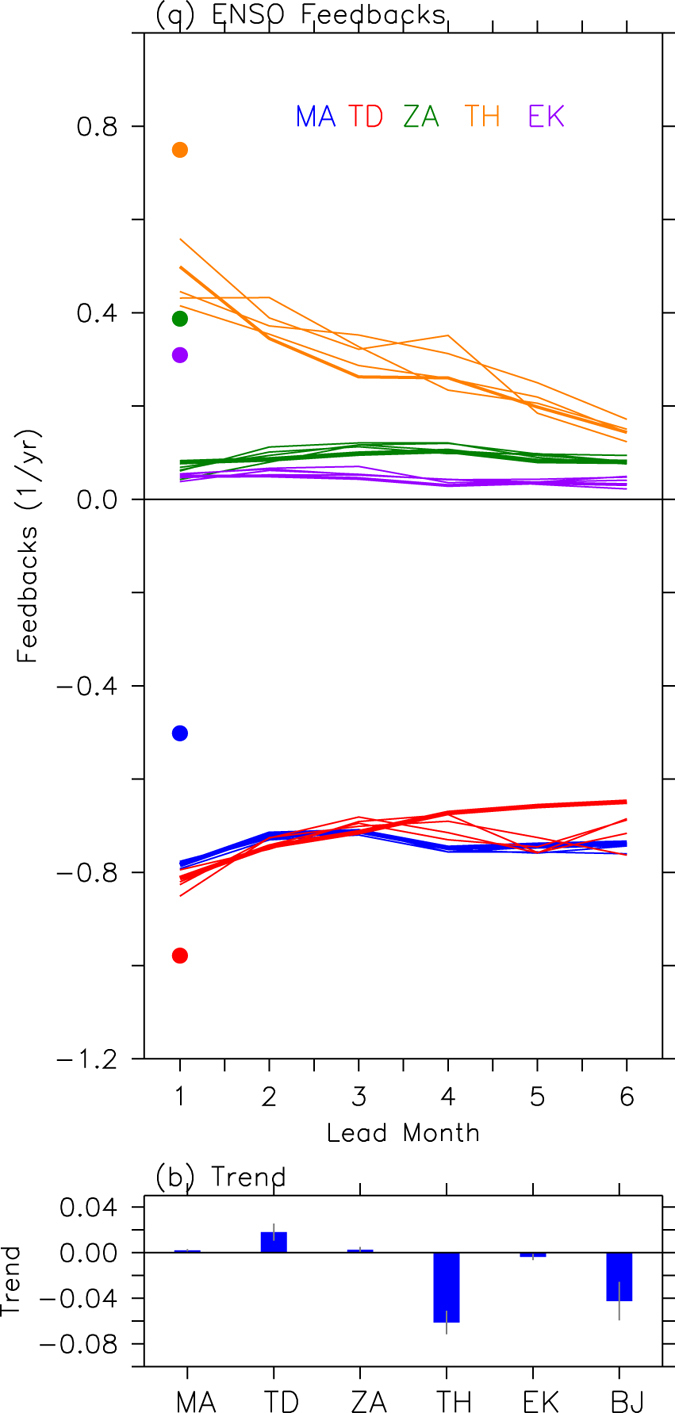
ENSO feedback errors. (**a**) Feedback terms in BJ index from predictions calculated at each forecast lead time. Blue (lines and dot) is mean advection damping (MA), red is thermodynamic damping (TD), green is zonal advective feedback (ZA), orange is thermocline feedback (TH), and purple is Ekman feedback (EK). Colored dots indicate results from observations. Solid lines indicate results from individual forecasts. (**b**) Trend (yr^−1^ lead mon^−1^) of each BJ index feedback term over forecast lead times. Blue bars show an average of all member forecasts and gray vertical lines show inter-ensemble member spread as estimated with standard deviation of trends from individual member forecasts.

Figure 3a shows the change in feedback terms contributing to the ENSO growth rate with different forecast lead times. First, the intensities of positive feedbacks [i.e., thermocline feedback (TH in Fig. 3a), zonal advective feedback (ZA in Fig. 3a), Ekman feedback (EK in Fig. 3a)] and thermodynamic damping terms (TD in Fig. 3a) are underestimated in the APCC CCSM3; this is also a discrepancy in other coupled climate models with respect to the observations^11^. Among positive feedback terms, the thermocline feedback term from the forecasts has a magnitude of 0.47 ± 0.05 yr^−1^ at the 1-month lead time, and the magnitude of the feedback term further decreases to 0.14 ± 0.02 yr^−1^ at a 6-month lead time, which is much smaller than that of the observations (0.75 yr^−1^). However, there are no clear decreasing tendencies of the other feedback terms with forecast lead times. This is evident in Fig. 3b, which shows the trend of each feedback term over the forecast lead times from the individual forecasts. The decreasing tendency of the BJ index (−0.04 ± 0.02 yr^−1^ lead mon^−1^) is mainly supported by that of the thermocline feedback (−0.06 ± 0.01 yr^−1^ lead mon^−1^). In contrast, the thermodynamic damping term, the trend of which has a magnitude of 0.02 ± 0.01 yr^−1^ lead mon^−1^, has an increasing tendency. Other terms (i.e., mean advection damping, zonal advective feedback, and Ekman feedback) have relatively small trend values. Therefore, the results suggest that ENSO amplitude forecast errors could be linked to those in the thermocline feedback.

**Figure 4:**
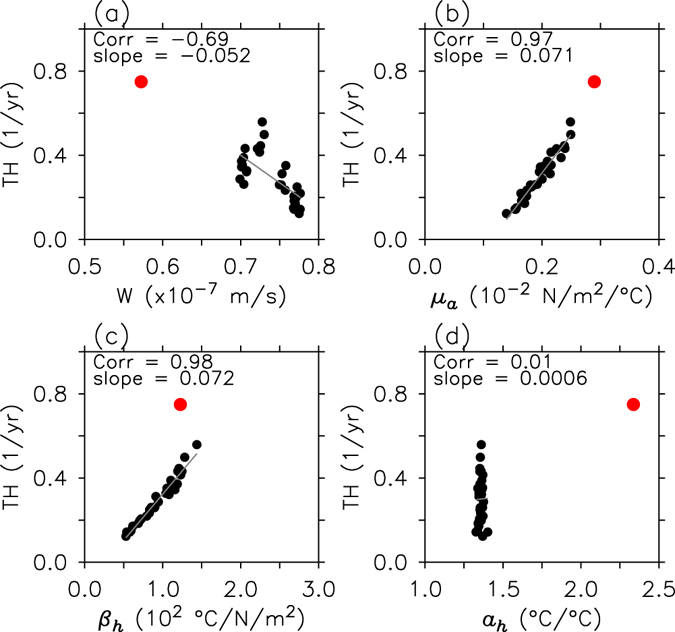
Factor for thermocline feedback error. Scatter plots of thermocline feedback versus its contributing factors, (**a**) W, (**b**) *μ*_*a*_, (**c**) *β*_*h*_, and (**d**) *a*_*h*_ from 1- to 6-month lead times. Black dots indicate individual member forecasts. The observed counterparts (red dots) for the thermocline feedback and contributing factors are also shown. Correlation coefficients and regression slopes, computed using samples from individual forecasts, are also shown.

Figure 4 shows scatterplots of thermocline feedback terms versus the associated mean states and response sensitivity coefficients from individual forecasts (black dots). In this figure, the correlation coefficients and regression coefficients are clearly shown to determine which factors dominate the error of the thermocline feedback term. Here, as for regression analysis, the terms relating to the thermocline feedback are normalized by their sample standard deviation in order to investigate the relative contribution of the factors to the thermocline feedback tendency over the forecast lead times. It was first identified that the underestimation of the thermocline feedback in the APCC CCSM3, with respect to the observations, is mainly due to the response sensitivity coefficients of the hindcast simulations, which are smaller than their counterparts in the observations. In contrast, mean upwelling is stronger in all forecasts than in the observations, which is related to the strong climatological zonal wind stress of the forecasts relative to that in the reanalysis (Supplementary Fig. 1a). Both the zonal anomalous thermocline slope response to wind forcing (*β*_*h*_) and the surface wind response to SST change (*μ*_*a*_) are most strongly correlated with a change in the thermocline feedback (*r* = 0.98 and 0.97, respectively). Regression analysis also shows that both response sensitivity coefficients (*β*_*h*_, *μ*_*a*_) have relatively large slopes with respect to thermocline feedback. Therefore, an increase in thermocline feedback errors with forecast lead times could be related to errors in the zonal thermocline slope response to wind change and the zonal wind response to SST change. The dominance of the thermocline slope response in the thermocline feedback has been suggested in previous numerical and observational studies^11, 21^, and some studies have speculated that the thermocline response errors are attributable to an upper ocean cold bias in the tropical Pacific^11, 20^. Furthermore, under a simple dynamical framework, the thermocline response depends on the mean thermocline depth and the upper ocean mean vertical stratification^22, 23^. Therefore, we investigate how the upper ocean temperature bias affects those oceanic mean states, and as a result, leads to thermocline response forecast errors.

**Figure 5:**
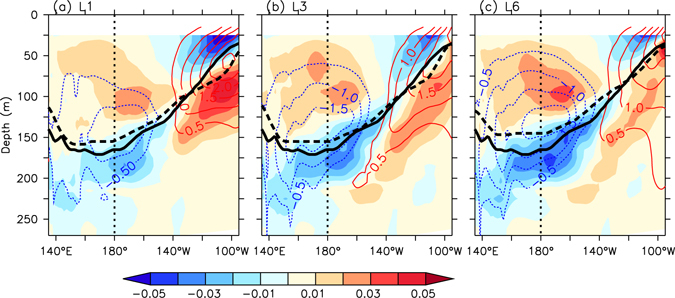
Tropical Pacific mean bias. Vertical cross section of difference between climatological mean ocean temperature (colored contour lines, contour interval = 0.5 °C, zero lines are removed) along the equator (2°S-2°N) and its vertical gradients (shades, °C m^−1^) over the hindcast periods (1983–2014) from the average of all member forecasts at (**a**) 1-, (**b**) 3-, and (**c**) 6-month lead times, and those from the GODAS reanalysis. The thermocline depth (depth of maximum temperature gradient within 0–400 m) from forecasts (thick black dashed-lines) and the observations (thick black solid-lines) are also superimposed.

Figure 5 shows the vertical cross-section of the differences between the climatological ocean temperatures (contours) along the equator from the average of all individual predictions at the 1-, 3-, and 6-month lead times and those from the ocean reanalysis (see Methods). In this figure the difference of the vertical gradients (shades) of the climatological ocean temperatures are also shown. It is found that the cold bias tends to become stronger, particularly around the thermocline in the western to central equatorial Pacific, and extends to the ocean surface with an increasing forecast lead time. It seems that the upper ocean cold bias starts in the equatorial Pacific thermocline. Previous study^29^ showed that the simulation of the Pacific tropical-extratropical water mass exchange through Pacific Subtropical Cell pathways can induce the thermocline bias in the CCSM3. In other words, the bias in the simulated thermocline may be due to the subduction of the colder water in the extratropical Pacific Ocean (Supplementary Fig. 1c). More studies including the investigation of the reason why the extratropical Pacific cold bias enhances with increase in forecast lead times would be performed in the future. This would provide some guidance to improve the coupled model.

Corresponding to the enhanced cold bias, the simulated thermocline depth becomes slightly shallower than that in the reanalysis. Around the eastern equatorial Pacific thermocline, a strong warm bias exists at early lead times; this is associated with a deeper thermocline depth in the forecasts than in the observation. However, the warm bias is gradually reduced with increasing forecast lead times. The increased cold bias around the thermocline in the western to central tropical Pacific, and the reduced warm bias in the eastern tropical Pacific, are associated with a weakening of the vertical temperature gradients (or thermal stratification) below the thermocline in comparison with the reanalysis. In contrast, the simulated vertical temperature gradients in the ocean above the equatorial thermocline become stronger than those in the ocean reanalysis with an increase in forecast lead time. Moreover, because the vertical temperature gradient difference between forecasts and observations, which is averaged from the surface to thermocline depth (Supplementary Fig. 2), and *β*_h_ is highly correlated with a magnitude of −0.93, model bias in the thermocline response is attributable to the bias in the mean vertical stratification. The increasing thermal stratification in the upper ocean may confine wind-forced momentum and as such prevent the wind force from being transferred effectively from ocean surface to the thermocline.

Regarding the dependence of *β*_*h*_ on the mean thermocline depth, because it is difficult to find the difference in the mean thermocline depth between 1-month to 6-month lead times in Fig. 5, the mean thermocline depth bias does not seem to be responsible for the thermocline slope response error.

In addition to the increased upper ocean stratification associated with the enhanced upper ocean cold bias, the meridional shape of the zonal wind stress response to the eastern equatorial Pacific SST change can also influence the thermocline feedback or the sensitivity of the zonal thermocline response. In other words, when the wind response is more confined to the equator, the thermocline feedback becomes stronger due to a strengthening of the equatorial thermocline response^30^. However, the wind stress response pattern in the APCC CCSM3 does not seem to affect the thermocline feedback because, with an increase in forecast lead times, the wind stress response tends to become meridionally narrower (Supplementary Fig. 3).

As for the decreased intensity of surface wind responses to SST change, indicating the role of the atmosphere in ENSO prediction errors, we could first relate this to an increase in the ocean surface cold bias, particularly in the central equatorial Pacific, with increasing forecast lead times (Supplementary Fig. 1b). In the tropics, where convective activity dominates the surface wind change, the wind response sensitivity to local SST changes may be weak under a relatively cold ocean background state because a large change in SST is needed to reach convective thresholds^31^. Second, the weak atmospheric response in the APCC CCSM3 might be due to a discrepancy in the parameterization scheme for deep atmospheric convection and the associated clouds, which controls atmospheric feedbacks during ENSO^32^.

## Summary and Discussion

This study used hindcast simulations made using the APCC CCSM3 to investigate the possible cause of forecast errors in simulating ENSO amplitude. The retrospective forecasts performed a 7-month integration for each of the 12 calendar months over a period of 33 years from 1982 to 2014. It was found that enhanced errors in ENSO amplitude with increased forecast lead times are well represented by those in the growth rate, as estimated using the BJ index. Therefore, BJ index analysis was used to reveal the cause of ENSO amplitude forecast errors.

This study identified that forecast errors in the thermocline feedback are most strongly associated with those in ENSO amplitude. These errors are mainly attributable to a weakened intensity of both the equatorial Pacific thermocline slope response to wind forcing and the surface wind response to SST forcing. Weakened atmosphere-ocean coupling is found to be closely related to upper ocean temperature bias in the tropical Pacific, which starts around the equatorial thermocline and intensifies with forecast lead times. The development of the upper ocean cold bias is associated with enhanced upper ocean thermal stratification error. Therefore, this study concludes that the tropical upper ocean cold bias and its associated vertical mean stratification error may be related to errors in the ENSO amplitude forecast made with the APCC CCSM3.

It seems that other state-of-the-art coupled models (see Figure 6 of ref. 11) also suffer from cold biases centered on the equatorial Pacific thermocline, and thus vertical thermal stratification is stronger than in the ocean reanalysis, leading to underestimated thermocline feedbacks with respect to the observations. This implies that the results of this study are potentially applicable to other coupled models. Therefore, this study strongly suggests that it is necessary to reduce the tropical Pacific upper ocean cold bias in coupled forecast models to provide better forecasts of the intensity of ENSO events.

As a concluding remark, this study mainly focused on the role of oceanic mean biases in ENSO-related feedback errors. Because the importance of the atmospheric component of ENSO growth has been highlighted in previous studies^32, 33^ and because the weak wind response may affect the thermocline slope response (correlation coefficient of *β*_*h*_ with *μ*_*a*_ is 0.93), the role of the atmospheric mean bias, which may be related to atmospheric feedback errors, should be investigated to identify whether the ocean or the atmosphere dominates ENSO amplitude.

## Methods

### Data and model

The atmospheric component of the APCC CCSM3 has a horizontal resolution of T85 (1.4° longitude × 1.4° latitude) and 26 vertical levels; the land surface component has the same horizontal resolution as the atmospheric component; the ocean component of the model has 40 vertical levels and a horizontal resolution of 1° in a longitudinal direction and 1° (1/3° near the equator) in the latitudinal direction; and the sea ice component has the same horizontal resolution as the ocean component.

Hindcast datasets of the APCC CCSM3 were produced by simulating a 7-month integration for each of the 12 calendar months in the 33 years from 1982 to 2014. Hindcast simulations were initiated from five different initial conditions in each month. Using a simple wind-ocean temperature nudging initialization method (similar to refs 34–36), the atmospheric and oceanic initial conditions were obtained as restart data from a coupled simulation where the simulated ocean temperatures and surface winds were strongly nudged toward their observations using a restoring time scale of five days and one hour, respectively. The observations were taken from the National Centers for Environmental Prediction (NCEP) Global Ocean Assimilation System (GODAS)^37^ datasets. The pentad mean GODAS datasets were interpolated into daily means using piecewise linear interpolation. To generate five ensemble member forecasts, hindcast simulations begin from the initial conditions of the atmosphere, land, and sea ice components in the last five days of each calendar month and all use the same initial oceanic conditions each month. The date of the initial ocean condition (when the forecast run starts) differs from month to month (25^th^ January, 24^th^ February, 26^th^ March, 25^th^ April, 25^th^ May, 24^th^ June, 24^th^ July, 23^rd^ August, 22^nd^ September, 27^th^ October, 26^th^ November, 26^th^ December) due to the availability of the pentad mean GODAS data. For example, the hindcast simulations initialized in August used the atmospheric initial conditions from the 27^th^, 28^th^, 29^th^, 30^th^, and 31^st^ of August, and the oceanic initial conditions from the 23^rd^ were used. Predictions for the month following the initial condition month are referred to as forecasts at a 0-month lead time. Therefore, the full 7-month integration produces forecasts from 0- to 6-month lead times.

All the monthly data at 1- to 6-month lead times from each of the ensemble member forecasts (i.e., individual forecasts) were reconstructed to obtain monthly datasets covering the period from 1982–2014 for each lead time. Using the reconstructed datasets, anomalies were then obtained by extracting the climatological seasonal cycle at each lead time from the individual forecasts. See Fig. 1 as an example of a time series of the reconstructed Niño 3.4 index at different lead times.

For ENSO forecast verification, the Niño 3.4 index (the area-averaged SST anomaly from 5°S-5°N and 170°–120°W) obtained from the hindcast simulations was compared to that obtained from the optimum interpolation SST version 2 (OISSTv2)^38^. Simulated ocean subsurface temperature was verified against the GODAS data set.

### BJ index formula

The BJ index is formulated as follows (see ref. 17 for a detailed formulation):

$$\begin{array}{ccc}2BJ & = & -({a}_{1}\frac{{\langle {\rm{\Delta }}\bar{u}\rangle }_{E}}{{L}_{x}}+{a}_{2}\frac{{\langle {\rm{\Delta }}\bar{v}\rangle }_{E}}{{L}_{y}})-{\alpha }_{s}+{\mu }_{a}{\beta }_{u}{\langle -\frac{{\rm{\partial }}\bar{T}}{{\rm{\partial }}x}\rangle }_{E}\\  &  & +{\mu }_{a}{\beta }_{w}{\langle -\frac{{\rm{\partial }}\bar{T}}{{\rm{\partial }}z}\rangle }_{E}+{\mu }_{a}{\beta }_{h}{a}_{h}{\langle \frac{\bar{w}}{{H}_{1}}\rangle }_{E}-\varepsilon ,\end{array}$$

where *BJ* denotes the BJ index, a greater magnitude of which implies that the tropical Pacific climate coupled system contributes to a stronger ENSO; *ε* is the damping rate related to the upper ocean adjustment, which is ignored^10^; $\bar{u}$, $\bar{v}$, $\bar{w}$, and $\bar{T}$ denote the climatological mean ocean zonal, meridional, and vertical velocity, and temperatures, respectively; *〈A〉*_E_ denotes area-averaged quantities over the eastern region of the equatorial Pacific basin; *L*_*x*_ and *L*_*y*_ represent the longitudinal and latitudinal length scales, respectively, of the eastern boxed region; and *H*_*1*_ (=50 m) is a mixed layer depth. The right-hand side of the BJ index formula, from left to right, denotes: mean advection damping, thermodynamic damping, zonal advective feedback, Ekman feedback, and thermocline feedback. Each positive feedback term is represented as a function of the tropical Pacific Ocean mean state and oceanic and atmospheric response sensitivity coefficients; the latter are computed using a least-squares linear regression from the approximated balance equations, namely, 〈*Q*〉_*E*_ = −*α*_*s*_〈*T*〉_*E*_, [*τ*_*x*_] = *μ*_*a*_〈*T*〉_*E*_, 〈*u*〉_*E*_ = *β*_*u*_[*τ*_*x*_] + *β*_*uh*_〈*h*〉_*W*_, ${\langle H(\bar{w})w\rangle }_{E}=-{\beta }_{w}[{\tau }_{x}]$, 〈*h*〉_*E*_ − 〈*h*〉_*W*_ = *β*_*h*_[*τ*_*x*_], and ${\langle H(\bar{w}){T}_{sub}\rangle }_{E}={a}_{h}{\langle h\rangle }_{E}$. *〈A〉*_W_ and [*A*] denote quantities that are area-averaged over the western boxed region and those averaged zonally over the equatorial Pacific basin (5°S-5°N, 121°E-82°W), respectively. $H(\bar{w})\,\,$is a step function, which is 1 (0) when $\bar{w} > 0\,(\bar{w} < 0)$, for ensuring that only upward vertical motion affects the mixed layer ocean temperature. The balance equations represent, respectively: the net anomalous heat flux (*Q*) change associated with change in an anomalous mixed-layer ocean temperature (*T*); an anomalous surface wind response (*τ*_*x*_) to an anomalous mixed-layer ocean temperature change; the response of mixed layer ocean zonal current anomalies (*u*), ocean upwelling anomalies (*w*), and an anomalous equatorial thermocline (*h*) slope to surface wind change; and an effect of anomalous thermocline depth changes on ocean subsurface (a depth of 50 m) temperature anomalies (*T*_*sub*_). The anomalous thermocline depth is estimated with an oceanic heat content anomaly, which is defined as the vertically averaged ocean temperatures from the ocean surface to a depth of 300 m.

GODAS datasets were used for the observed BJ index. The boxed regions for the APCC CCSM3 and reanalysis (i.e., GODAS) were determined from the spatial pattern of heat content anomalies associated with their own leading ENSO mode (also see ref. 17 for a detailed methodology for determining the boxed region). The eastern boxed region (i.e., *〈A〉*_E_) for the APCC CCSM3 is defined as 5°S-5°N, 171°W-82°W. The longitude of the western edge of the eastern boxed region is an average (171°W ± 3°) of those from all members and all forecast lead times. When we used different longitudes from each member and each lead time, similar results were obtained. For the GODAS datasets, the eastern region is defined as 5°S-5°N, 173°W-82°W. The western boxed region (i.e., *〈A〉*_W_) covers from 121°E to the longitude of the western edge of the eastern boxed region.

### Data availability

The datasets generated during and/or analyzed during the current study are available from the corresponding author on reasonable request.

## Electronic supplementary material

Supplementary Information
