# Supplementary material for: Mean Bias in Seasonal Forecast Model and ENSO Prediction Error
Source: Sci Rep. 2017 Jul 20;7:6029. doi: 10.1038/s41598-017-05221-3 (PMC5519555; doi:10.1038/s41598-017-05221-3)
Supplement: Supplementary file 1 — Supplementary Information [file 41598_2017_5221_MOESM1_ESM.pdf]

Supplementary Information for

Mean Bias in Seasonal Forecast Model and ENSO Prediction Error

Seon Tae Kim[1], Hye-In Jeong[1], and Fei-Fei Jin[2]

[1]Climate Prediction Department, APEC Climate Center, Busan, South Korea.

[2]Department of Atmospheric Sciences, University of Hawaii at Manoa, Honolulu, HI, USA.

Corresponding author: Seon Tae Kim (seontae.kim@apcc21.org)

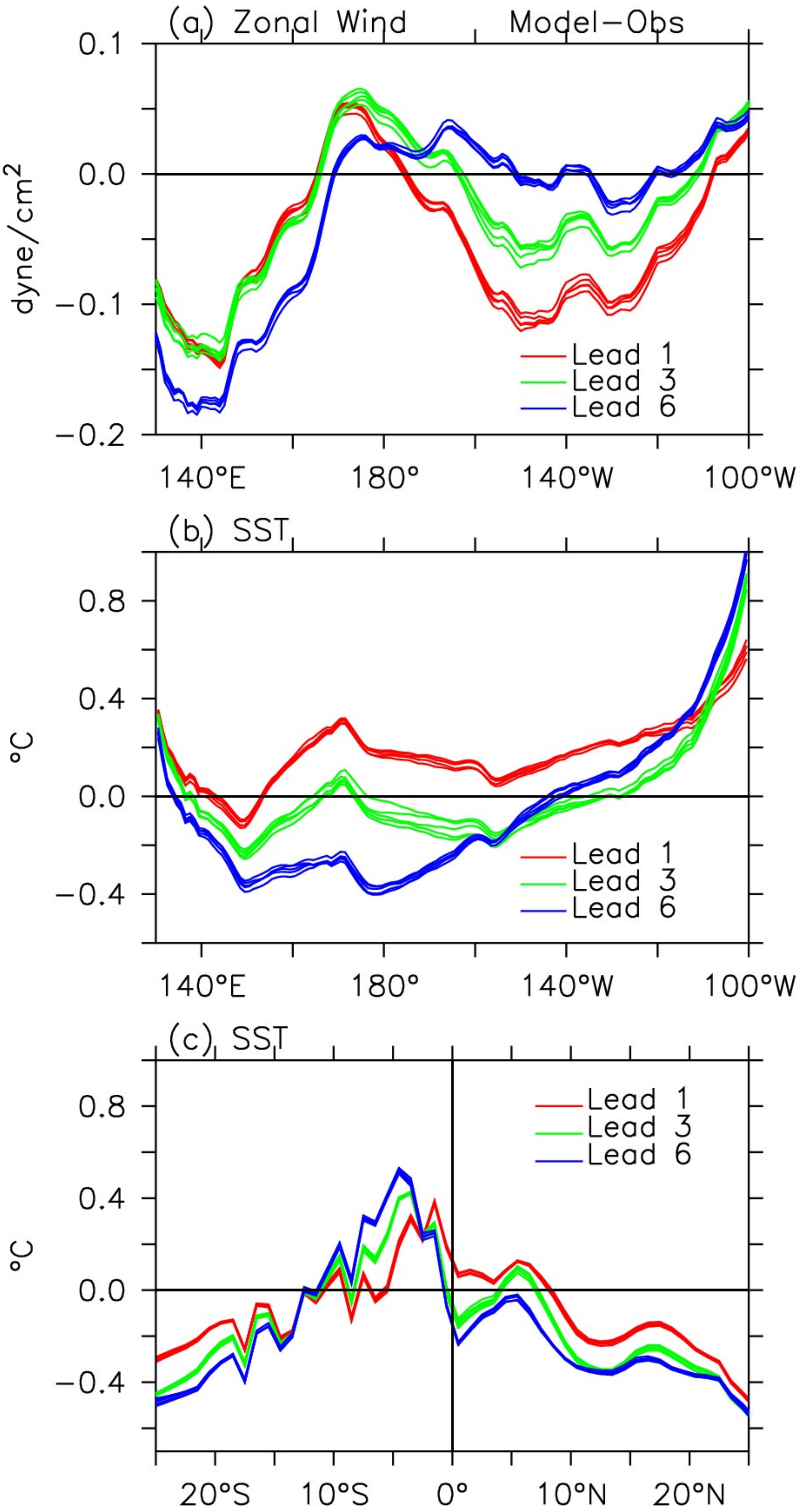
(a) Zonal Wind
Model−Obs
dyne/cm^2
0.1
0.0
−0.1
−0.2
Lead 1
Lead 3
Lead 6
140°E
180°
140°W
100°W
(b) SST
°C
0.8
0.4
0.0
−0.4
Lead 1
Lead 3
Lead 6
140°E
180°
140°W
100°W
(c) SST
°C
0.8
0.4
0.0
−0.4
Lead 1
Lead 3
Lead 6
20°S
10°S
0°
10°N
20°N

Supplementary Figure 1. Climatological wind and SST bias. Difference of climatological (a) zonal wind stress and (b) SST along the equator (2°S-2°N), and (c) SST averaged over 135°W-100°E between forecasts (red lines for 1-month lead, green lines for 3-month lead, and blue lines for 6-month lead forecasts) and observations. For observations, we use OISST and GODAS reanalysis datasets.

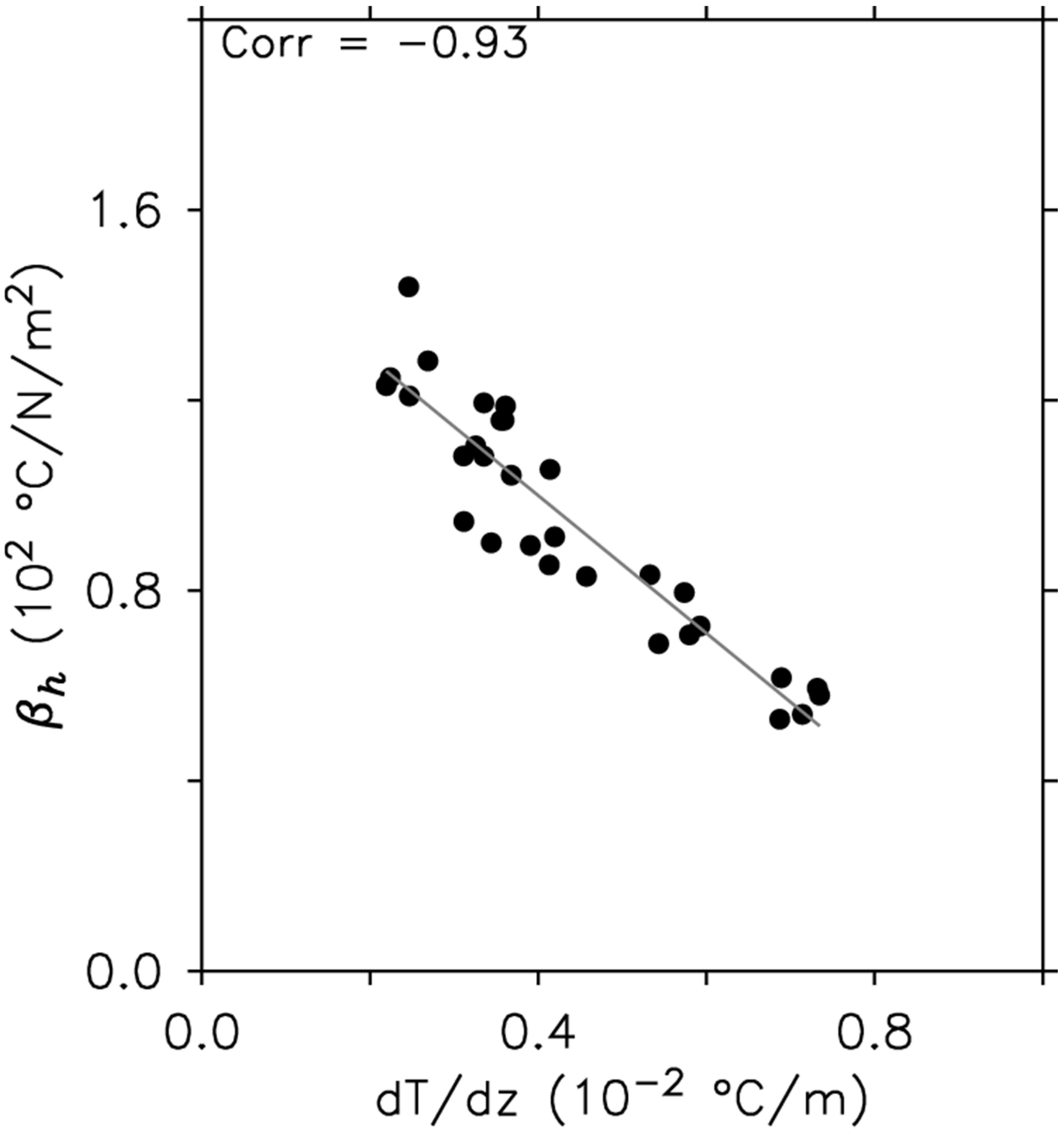

Supplementary Figure 2. ***β_h* and vertical thermal stratification of upper ocean**. Scatter plot of β_h versus mean vertical temperature gradient difference between individual member forecasts at 1- to 6-month lead times and observations. The mean vertical temperature difference is computed at each grid point (see Fig. 5 in main text) and then averaged vertically from surface to thermocline depth and horizontally over the region of 120°E-80°W, 2°N-2°S.

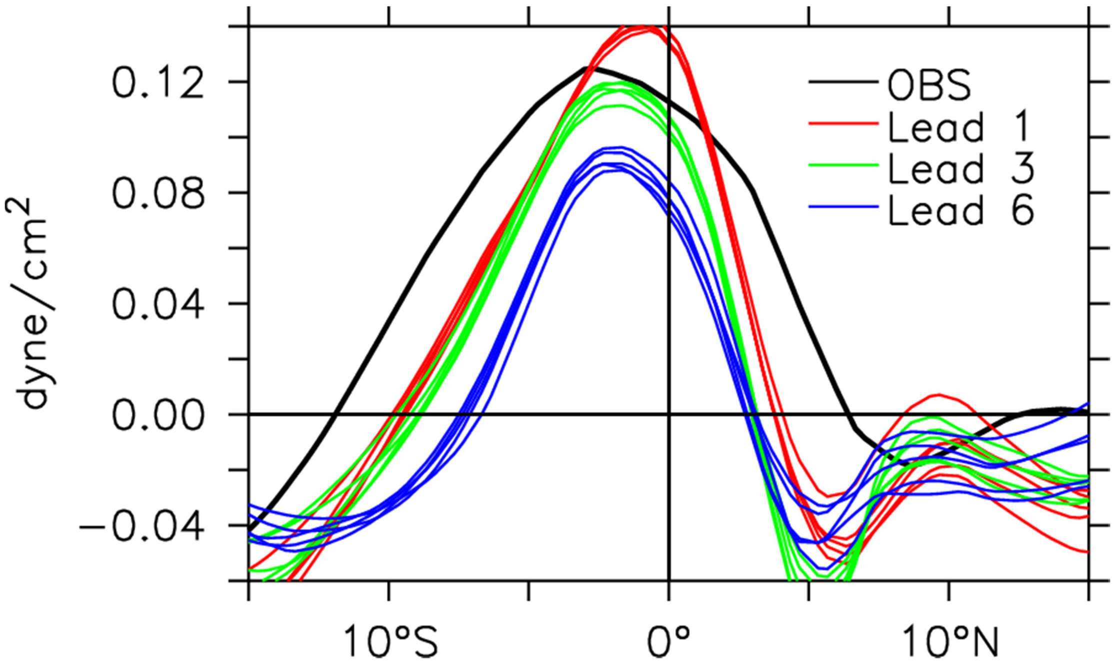

Supplementary Figure 3. Meridional shape of zonal wind response to SST forcing. Regression coefficients, which are zonally averaged over the Niño 4 regions (160°E-150°W), between zonal wind stress anomalies and ENSO-related SST change. ENSO-related SST change is represented by time series of principal components of the first leading EOF mode for tropical Pacific SST anomalies.
